# Lessons from oncology: Can cancer lead the way in resistance management?

**DOI:** 10.1093/emph/eoag023

**Published:** 2026-09-17

**Authors:** Silvie Huijben, Ernest Boampong, Harley Richker, Beckett Sterner, Carlo C Maley

**Affiliations:** The Center for Evolution & Medicine, School of Life Sciences, Arizona State University, Tempe, AZ, USA; Simon A. Levin Mathematical, Computational and Modeling Sciences Center, Arizona State University, Tempe, AZ, USA; The Center for Evolution & Medicine, School of Life Sciences, Arizona State University, Tempe, AZ, USA; The Center for Evolution & Medicine, School of Life Sciences, Arizona State University, Tempe, AZ, USA; The Arizona Cancer Evolution Center, Arizona State University, Tempe, AZ, USA; Biodesign Center for Biocomputing, Security and Society, Arizona State University, Tempe, AZ, USA; School of Life Sciences, Arizona State University, Tempe, AZ, USA; The Center for Evolution & Medicine, School of Life Sciences, Arizona State University, Tempe, AZ, USA; The Arizona Cancer Evolution Center, Arizona State University, Tempe, AZ, USA; Biodesign Center for Biocomputing, Security and Society, Arizona State University, Tempe, AZ, USA

**Keywords:** resistance evolution, cancer, resistance management, public health, agriculture, drug resistance

## Abstract

The evolution of resistance to chemical interventions is a widespread challenge in medicine and agriculture, affecting bacteria, viruses, parasites, cancer, pests, and weeds. Many resistance management strategies have been proposed, but empirical comparisons are rare, especially in large-scale systems like agriculture and public health, where populations are difficult to replicate, and interventions cannot easily be tested experimentally. Cancer offers a unique opportunity to study resistance evolution. Because evolution occurs within individual patients, resistance strategies can be tested directly in clinical trials, with each patient serving as an independent replicate. Combined with diverse therapies and experimental models, this makes oncology a powerful system for studying therapeutic resistance. Whether insights from cancer apply to other systems remains an open but testable question. Addressing this could reveal general principles of resistance evolution and help unify currently fragmented approaches to resistance management across disciplines.

The twentieth century saw unprecedented expansion in the use of drugs and pesticides, enabling significant improvements in crop production and the treatment of once-deadly diseases. However, these gains are increasingly undermined by evolutionary forces that drive pathogens and pests to become ever more difficult to control with once-useful chemistries. The emergence of resistance to pesticides in agriculture, antimicrobials and antivirals in medicine, and therapies in cancer has become a global challenge that threatens the effectiveness of some of our most important tools. For example, antimicrobial resistance was predicted to cause up to 10 million deaths annually by 2050, exceeding predicted cancer death rates [1]. Furthermore, resistance to first-line antiretroviral drugs has risen sharply, leading to HIV treatment failures in parts of sub-Saharan Africa [2], and malaria treatment is down to only one widely effective drug: artemisinin-based therapies, with partial resistance already established in Southeast Asia [3]. Herbicide-resistant weeds devastate crop yields worldwide [4], while insecticide-resistant pests such as the diamondback moth [5] and the western corn rootworm [6] pose major challenges to crop production. Finally, in oncology, resistance to systemic therapies remains a major barrier to durable cancer cures [7]. The financial burden of resistance is staggering across health care and agriculture. The World Bank has estimated that antimicrobial resistance could increase health costs from $330 billion to $1.2 trillion [8], and the diamondback moth alone is estimated to cost the world economy $4–5 billion annually in lost brassica vegetables and oilseed crops [5]. The substantial financial burden on agriculture has created strong incentives for the industry to develop effective resistance management strategies, supported by regulatory stewardship programs and, in some jurisdictions, requirements for integrated pest management and resistance management plans [9]. As a result, agriculture is often more advanced in this area, offering lessons that other fields, such as vector control [10] and oncology [11], may draw upon. Here, we reverse this perspective and argue that oncology may be able to contribute to the field of resistance management beyond cancer.

Resistance to chemical interventions arises through natural selection: variants carrying heritable changes that confer a survival advantage in the presence of the drug or pesticide are more likely to reproduce and pass on those traits. Yet the evolutionary dynamics are rarely this straightforward. Resistance mutations often carry fitness costs, making resistant variants less competitive in the absence of treatment (e.g. [12, 13]). These opposing forces (positive selection under treatment and negative selection due to fitness costs) create opportunities for resistance management strategies that reduce the selective pressure for resistance while maintaining effective control of the pathogens, pests, or cancer cells. A variety of resistance management strategies have been proposed and implemented across different fields. Mixtures (or combination therapies) deploy multiple active ingredients simultaneously, usually with different modes of action, reducing the probability that a single mutant carries all the necessary mutations to survive. Rotations and mosaics increase environmental heterogeneity by varying the active ingredient over time (rotations or cycling) or across space (mosaics). These three approaches (mixtures, rotations, and mosaics) represent the most widely applied resistance management strategies in both agriculture and medicine [14]. In oncology, a novel approach known as adaptive therapy has emerged: treatment is dynamically adjusted based on tumor growth, aiming to maintain control while avoiding the intense selective pressure associated with continuous high-dose treatment [15, 16]. Comparable concepts are being explored in infectious disease and agriculture. For example, dose-moderated antimalarial treatment has been investigated as a means of slowing resistance evolution [17], while in crop systems, high-dose/refuge strategies leave part of a field untreated to reduce selection [18]. Adaptive therapy, dose moderation, and high-dose/refuge all share a common principle: shifting the goal from eradication to population control by using only as much chemical input as necessary for the intended clinical or economic outcome. Finally, the most effective long-term resistance management is to reduce or eliminate reliance on chemical interventions altogether, replacing them with mechanical, biological, or preventive methods that suppress populations without driving chemical resistance.

The evidence required to determine which resistance management strategy is most effective in reducing the emergence or spread of resistance while maintaining intended treatment goals is hard to obtain [14]. Predictive mathematical models provide powerful tools for designing effective resistance management strategies. Across fields ranging from antibiotics to insecticides to cancer therapies, models have been developed and applied to evaluate the effectiveness of various resistance management strategies on allele frequencies at the population level [14, 19, 20]. Though useful, mathematical models cannot fully capture the complexity of real-world dynamics and cannot replace the need for empirical data on the efficacy of resistance management strategies. For large-scale complex evolving systems, like in agriculture and public health, large field-scale studies are required to evaluate how different interventions shape long-term evolutionary outcomes. Designing such studies requires multiple replicate field sites with comparable ecological, environmental, and epidemiological conditions, spaced far enough apart to minimize the impact of gene flow between them. These studies are logistically challenging and costly to conduct. For instance, the only large field-scale evidence of insecticide resistance management for mosquito control is from a three-year study in Mexico [21, 22]. However, this study failed to show that insecticide rotations and mosaics were much better in resistance management in comparison to single-insecticide application. In the case of antibiotics, assessing the evolutionary impact of resistance management strategies requires coordinated trials across multiple wards and hospitals. Such large-scale studies are resource-intensive and remain the exception rather than the norm [23]. In a large study involving seven ICUs across five European countries, no difference in antibiotic resistance was observed comparing rotations versus mosaics of antibiotics [24]. High-dose/refuge strategies have not been evaluated in multi-year randomized field experiments, presumably due to the difficulty of coordinating trials across independent farms and the economic risks for growers. Instead, the strongest evidence comes from large, spatially explicit quasi-experiments, which show prolonged delays, or even absence, of resistance evolution in areas with high-dose/refuge, compared with rapid resistance emergence where such strategies were lacking [25].

Resistance evolution is comparatively easier to study in cancer. Evolution is contained within a single patient, making the patient the natural unit of replication for testing treatment strategies, rather than an entire field site or hospital ward. Of course, this comes with its unique practical and ethical challenges, such as patient heterogeneity, treatment toxicity, and the limited ability to directly observe tumor evolution. Nevertheless, oncology has made substantial strides in testing evolutionary treatment strategies, including pilot studies demonstrating that adaptive therapy can slow resistance [16]. A further distinction is that most cancer patients begin treatment with tumor lineages that have no prior evolutionary history of selection by the drug: while mutations may be present at baseline at low frequencies, they have not yet been selected for by exposure to the treatment. By contrast, in agriculture and public health, evolution is cumulative: pesticides and drugs have been exerting selective pressure for decades, so resistance alleles are already widespread, sometimes at very high frequency, at the onset of treatment. The consequence is that in agriculture and public health, chemical tools are lost irreversibly over time, whereas in cancer, each new patient effectively represents a fresh evolutionary starting point. Over the past half-century, numerous new antimicrobial agents, pesticides, and anticancer drugs have been developed. In oncology, this has steadily expanded the therapeutic arsenal. However, in agriculture and public health, the introduction of new chemistries has not kept pace with resistance, leaving the available toolkit largely stagnant or even diminished over recent decades (Fig. 1).

**Figure 1:**
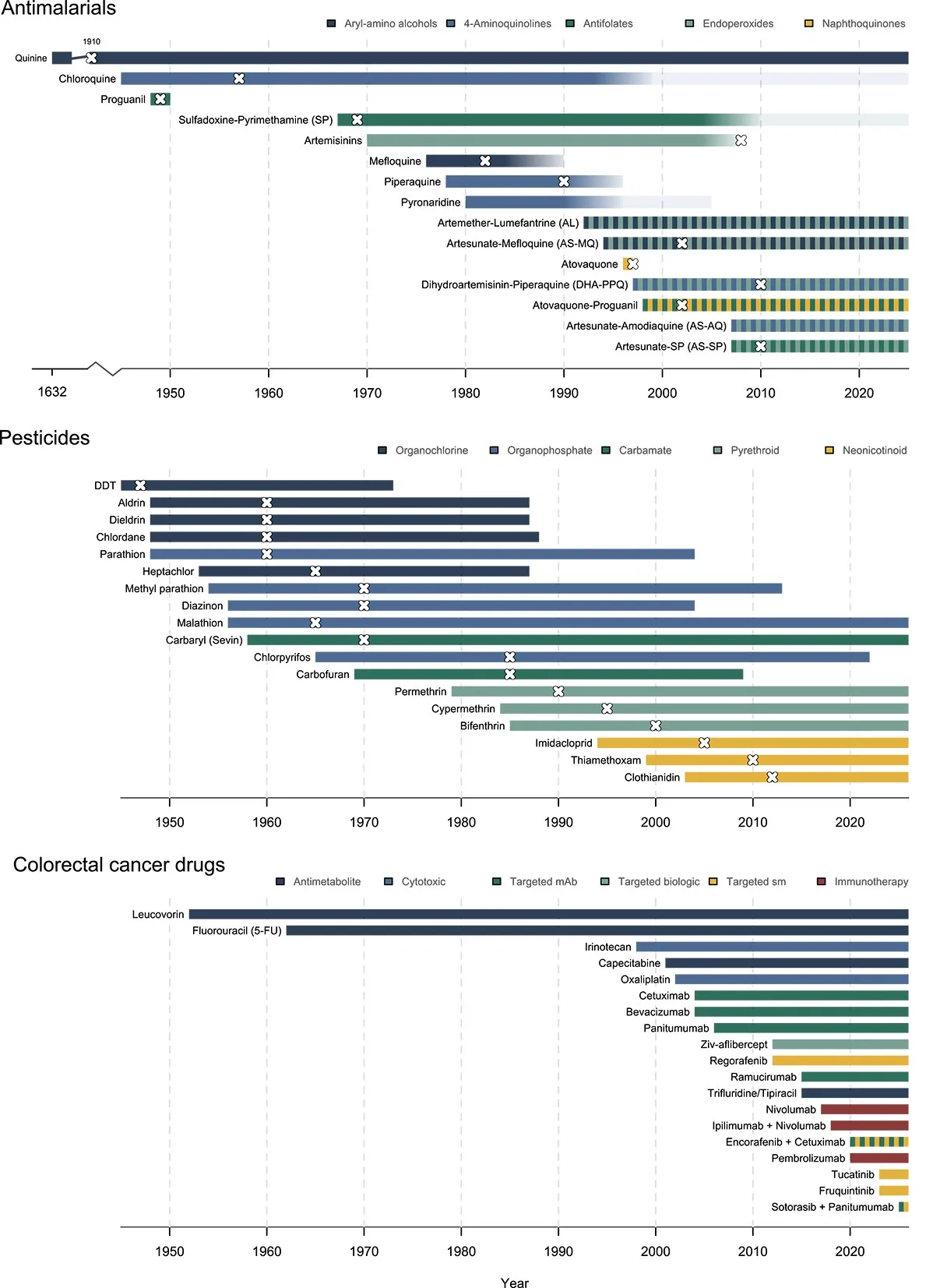
Timelines of the introduction and duration of use of chemical interventions for malaria treatments (top), insect pest control (middle), and colorectal cancer therapy (bottom); bar colors denote drug or pesticide class (antimalarials and pesticides) or group (cancer therapy); faded colors show drugs still in use for limited purposes; for combination treatments, alternating colors indicate active ingredients of different classes or groups used simultaneously; white cross symbols mark the first reported emergence of resistance to each compound; antimalarial data adapted from World Health Organization [48], pesticide data from the U.S. Environmental Protection Agency (EPA) database [https://www.epa.gov] with resistance data from the arthropod pesticide resistance database [https://www.pesticideresistance.org/], colorectal cancer data were obtained from the U.S. Food and Drug Administration (FDA) approval database [https://www.fda.gov]; targeted mAb = targeted monoclonal antibody; targeted sm = targeted small molecule.

Another factor that may make resistance management in cancer somewhat more tractable than in other systems is that cancer cells reproduce predominantly asexually. The distinction matters because asexual populations lack recombination. While bacteria also reproduce asexually, many are capable of recombination via the horizonal transfer of DNA from one bacterium to another, which may even cross species boundaries [26]. Thus, although important exceptions exist, recombination is a complexity that is part of the majority of resistance-prone systems, such as malaria parasites, weeds, pest insects, many bacteria, and even viruses. Recombination can accelerate resistance by bringing together beneficial mutations from different backgrounds, such as resistance that requires mutations in multiple genetic backgrounds, resistance to combination treatments requiring multiple mutations, or resistance alleles that are linked to compensatory mutations restoring fitness [12, 26, 27]. As such, resistance management strategies that rely on delaying the accumulation of multiple resistance mutations, or exploiting fitness costs may be less effective in recombining populations. However, it is important to note that recombination could also slow resistance evolution by breaking apart beneficial loci, particularly at the early stages of evolution [27], and the overall effect remains to be determined. A further consequence of asexual reproduction is susceptibility to Muller’s ratchet, with the irreversible accumulation of deleterious mutations potentially leading to mutational meltdown [28]. This concept originates from asexual organisms, but applies to asexually reproducing, non-recombining somatic cancer cells as well, which have evolved strategies to buffer against these deleterious effects [29, 30]. There is some evidence, however, that cell fusion and other mechanisms of horizontal gene transfer in cancers might provide some of the genomic benefits of sexual reproduction [31]. Phenomena like copy-neutral loss of heterozygosity may ameliorate Muller’s ratchet by removing deleterious mutations, but the extent of those phenomena remains an open question in cancer.

To be fair, cancer treatment also faces challenges that are absent in other systems. Most notably, the target for cancer treatment is a human cell. As a result, the therapeutic window is often narrower than for antimicrobials or pesticides that exploit fundamental differences between host and target, meaning that relatively modest increases in tumor cell resistance compared with surrounding normal tissues may be sufficient to limit treatment. Therefore, there are fewer exploitable vulnerabilities and consequently fewer distinct drug classes, as well as high levels of toxicity from cancer drugs [32, 33]. Additionally, the human genome is, with its ~3 Gb [34], orders of magnitude larger than those of bacteria (0.5–10 Mb [35]), RNA viruses (10–30 Kb [36]), and most pest and vector species (e.g. diamondback moths: 328 Mb [37]; malaria mosquito: 264 Mb [38]). The exception is weeds, where plant genomes vary widely in size but can be larger than those of humans (common pest weeds: 270 Mb–4.4 Gb [39]). Interestingly, cancers mainly evolve through the inactivation/deletion and activation/amplification of genes that are already in the human genome, with the occasional generation of new genes by splicing together pieces of two genes via translocations [40]. They also tend to activate mobile genetic elements through hypomethylation, which probably drives additional genomic instability [41]. While many factors shape evolutionary potential, all else being equal, a larger genome means more mutational opportunities that may facilitate adaptation, either by conferring drug resistance directly, or by restoring and improving the fitness of the tumor cell. This may explain why mixtures of cancer drugs typically provide only limited benefit over single drugs (e.g. [42, 43]). In addition, the stakes in cancer therapy are high: human lives. This has understandably led to conservatism in medical research, requiring extensive pre-clinical evidence as well as pilot clinical trials before the large (and expensive) definitive randomized controlled trials can be done. Good evidence-based medicine requires positive results from such trials before clinical practice can change.

So, is cancer research better positioned to test strategies to control resistance evolution? The answer is yes, at least in some contexts. Continued research and development are steadily expanding the anticancer therapeutic arsenal. Unlike pesticides and antimalarial drugs, whose utility can decline over time because resistance accumulates across populations, anticancer drugs generally remain available because resistance evolves independently within each patient (Fig. 1). Of course, individual cancer drugs may still be replaced because of toxicity, superior alternatives, or narrower clinical indications. However, unlike pesticides and many antimicrobial agents, they do not typically become useless through the cumulative spread of resistance across the treated population. This also fundamentally changes the incentives for resistance management. Unlike pesticides and antimicrobial drugs, where resistance evolution creates a shared societal problem requiring stewardship to preserve efficacy for future users, cancer presents a different incentive structure. In antimicrobial treatment, the incentive to cure an individual patient may conflict with the incentive to prevent the spread of resistance. In cancer, by contrast, these incentives are more aligned: successful clinical outcomes depend on managing resistance, without conflicting with preserving the patient’s life or the drug’s efficacy for future patients. There is a vast diversity of animal and *in vitro* (including organoid) models for pre-clinical testing in cancer [44]. Indeed, novel resistance management strategies are being tested and implemented. One simple approach is to reduce the intensity of selection for resistance by reducing the dose of drugs. In theory, this should delay the time to progression and relapse, while also reducing the toxicity of the treatment. De-escalation clinical trials, where aggressive end-of-life treatment may be replaced with early palliative care, have received recent interest and have even been shown to extend survival [45]. Patient survival rates for many cancers have increased steadily over recent decades, although these effects are partially attributable to improved diagnosis (e.g. [46, 49]). By contrast, there are numerous examples where progress in other fields has been less encouraging. Global malaria control has stagnated, with incidence and mortality rising again in recent years [48]. After the initial success of antibiotics, mortality due to antibiotic-resistant infections is rising, particularly among the elderly, potentially threatening any of the gains that have been made [49]. In agriculture, escalating resistance to herbicides has led to increased use of herbicide in the environment, yield losses, and rising production costs [50]. In this light, oncology may provide the strongest opportunities to develop and empirically test resistance management strategies. Crucially, cancer research benefits from the ability to test different resistance management strategies directly in clinical trials, an opportunity rarely available in other systems. Whether insights from oncology will translate effectively to other systems remains an open and important but testable question. Their generality will likely depend on biological and operational characteristics of the system, including the extent of prior selection, recombination, fitness costs, the feasibility of monitoring evolving populations, and the ability to experimentally compare alternative management strategies. Determining which general principles of resistance evolution are universal and which are system-specific represents an important direction for future research. However, the field of oncology may be in the position to not only advance its own field but also to lead the broader science of resistance evolution. In most other settings, we can only wish we could go back in time, armed with today’s knowledge and tools, to apply better resistance management from the beginning of chemical usage. Oncologists, uniquely, can approach each new patient as a novel starting point for understanding evolutionary dynamics, and, with that, have the rare opportunity to shape the future of resistance management across disciplines.

## Data Availability

No new primary data were generated for this article. Data underlying Figure 1 were derived from publicly available sources, including the World Health Organization, the U.S. Environmental Protection Agency, the Arthropod Pesticide Resistance Database, and the U.S. Food and Drug Administration, as detailed in the Figure 1 legend.
